# Bioinformatics analysis and experimental verification of TIGD1 in non-small cell lung cancer

**DOI:** 10.3389/fmed.2024.1374260

**Published:** 2024-04-08

**Authors:** Lingchun Xia, Zhuofan Yang, Mingming Xv, Guohui Wang, Yaxin Mao, Yihan Yang, Jian Tang

**Affiliations:** ^1^Department of Thoracic Surgery, The First Affiliated Hospital, Jiangxi Medical College, Nanchang University, Nanchang, China; ^2^Department of Urology, Tianjin Medical University General Hospital, Tianjin, China; ^3^Jiangxi Medical College, Nanchang University, Nanchang, China; ^4^Jiangxi Institute of Respiratory Disease, The First Affiliated Hospital of Nanchang University, Nanchang, China; ^5^Pulmonary and Critial Care Medicine, Jiangxi Hospital of China-Japan Friendship Hospital, Nanchang, China

**Keywords:** oncology, lung cancer, gene therapy, bioinformatics analysis, drug sensitivity

## Abstract

**Introduction:**

Non-small cell lung cancer (NSCLC) is a prevalent respiratory system tumor. Triggered transposable element derivative 1 (TIGD1) exhibits significant overexpression in various tumor cells and tissues, suggesting its involvement in cancer progression.

**Methods:**

Clinical data and gene expression profiles of lung adenocarcinoma were collected from TCGA, UCSC XENA, and GEO databases. Computational techniques and empirical studies were employed to analyze the role of TIGD1 in NSCLC. Cellular experiments were conducted using the H1299 cell line, including RNA interference, cell viability assays, quantitative PCR, wound-healing assays, western blotting, and plate clone formation assays.

**Results:**

Bioinformatics analysis revealed TIGD1’s potential as a biomarker for diagnosing and predicting lung cancer. It also indicated promise as a target for immune-related therapy and targeted drug therapy. Cellular studies confirmed TIGD1’s involvement in cancer cell proliferation, invasion, and migration. Furthermore, an association between TIGD1 and the PI3K/AKT signaling pathway was suggested.

**Discussion:**

The findings suggest that TIGD1 plays a vital role in NSCLC progression, making it a potential diagnostic biomarker and therapeutic target. The association with the PI3K/AKT signaling pathway provides insights into the underlying molecular mechanisms. Integrating computational analysis with empirical studies enhances our understanding of TIGD1’s significance in NSCLC and opens avenues for further research into targeted therapies.

## Introduction

Lung cancer is recognized globally as the predominant cause of death related to cancer, and non-small cell lung cancer (NSCLC) accounts for approximately 85% of these instances. NSCLC is notorious for its high mortality rate, limited effectiveness of treatment, and a propensity for strong metastasis (1, 2). Even with advanced treatment approaches available, the prognosis for many NSCLC patients remains bleak, often resulting in low survival rates (3, 4). Thus, delving deeper into the molecular mechanisms underlying lung cancer and identifying new potential therapeutic targets are crucial for advancing NSCLC diagnosis and treatment strategies.

The gene known as Triggered transposable element derivative 1 (TIGD1), which is exclusive to humans, encodes a protein. This protein is distinguished by the presence of three pfam domains: a DNA-binding HTH domain located between amino acids 9 and 60, a HTH CenpB-type DNA-binding domain spanning amino acids 80–147, and a DDE endonuclease domain that extends from amino acid 216–403 (5). Belonging to the TIGD gene family, TIGD1’s protein shares notable structural and functional characteristics with the mammalian centromere protein B (CENP-B) and exhibits a significant relationship with cell cycle-related proteins (6). Despite this, the precise biological roles of TIGD1 are still largely unexplored (7). Previous studies have demonstrated the potential pivotal function of TIGD1 in cancer cell proliferation, invasion, and migration using bioinformatics techniques. It has been reported that the expression changes of TIGD1 are particularly significant during the occurrence of liver cancer, suggesting its potential involvement in the development of liver cancer (7). Moreover, TIGD1 exhibits heightened expression across several cancer types, such as colorectal, lung, and pancreatic cancers. Notably, a correlation exists between elevated TIGD1 expression and adverse disease outcomes in individuals suffering from breast, liver, lung, and gastric cancers (8). Recent research indicates that TIGD1 also has a significant impact on immune response and chemotherapy response. For example, in the study of oral squamous cell carcinoma, researchers found that TIGD1 regulates dendritic cell activity by activating the IL-17 signaling pathway, thereby promoting the occurrence and progression of oral squamous cell carcinoma. In previous studies on ovarian cancer, it was observed that TIGD1 had an impact on the response of ovarian cancer patients to platinum-based chemotherapy (9). In their research, Zou and colleagues combined bioinformatics techniques with *in vitro* cellular studies to identify TIGD1 as a standalone prognostic indicator in colon cancer. The study suggests that TIGD1 accelerates the transition of cancer cells from the G1 to the S phase by triggering various colon cancer signaling pathways, such as Wnt/B-catenin, E-cadherin, N-cadherin, Bcl-2, BAX, CDK6, and cyclin D1. This process facilitates a smoother progression for cancer cells while simultaneously inhibiting apoptosis (10). Furthermore, another study has observed that TIGD1 can potentially augment cell death caused by copper toxicity in colorectal cancer cells, by elevating the concentrations of Cu ions (11). These studies have revealed TIGD1’s considerable potential as a marker in tumor identification and as a pivotal target in the realm of immunotherapy. However, further in-depth investigation is required to determine its specific clinical translation value.

The exact function and operational mechanism of TIGD1 in non-small cell lung cancer (NSCLC) remain ambiguous. Consequently, this research is focused on examining the effects of TIGD1 on NSCLC cell growth and mobility, and on delving into its possible mechanism of action.

## Materials and methods

### Download and preprocess TCGA and GEO data

We sourced clinical information and gene expression data from The Cancer Genome Atlas (TCGA), UCSC XENA, and the Gene Expression Omnibus (GEO) databases. In this study, we utilized lung adenocarcinoma (LUAD) patient data obtained from the TCGA database. Our dataset comprises mRNA sequencing (RNA-seq) data and clinical information for 515 patients with lung adenocarcinoma (LUAD). It includes 515 LUAD tumor tissue samples and 59 adjacent normal tissue samples. Using the R programming language, we normalized the gene expression data, converting it from the FPKM (fragments per kilobase million) format to the TPM (transcripts per kilobase million) format. This conversion ensured the data’s uniformity and precision, setting the stage for our subsequent analyses.

### UALCAN analysis

UALCAN^1^ is an extensive and user-friendly online platform. We collected RNA sequencing (RNA-seq) data and clinical information for 31 cancer types to conduct a detailed comparison of gene expression patterns between tumor and normal tissues. This analysis also aimed to identify variations in gene expression across different tumor stages and other clinicopathological characteristics. Furthermore, we specifically examined the expression levels of the TIGD1 gene, considering various clinical parameters, including tissue type and clinical stage, to understand its role in cancer progression better.

### Survival analysis

The KM plotter database is a widely recognized public database for analyzing gene expression and disease prognosis.^2^ In this study, we employed this database to explore the association between TIGD1 expression levels in lung adenocarcinoma (LUAD) and the overall survival rates of the patients. For evaluating the importance of survival disparities between two groups, we utilized the log-rank test. Furthermore, we accounted for potential confounding factors that could impact survival analysis by analyzing the clinical data from the LUAD dataset in the TCGA database. These aspects encompassed clinical grading, pathological staging, among others, thereby guaranteeing the precision and trustworthiness of the outcomes derived from our analysis.

### Functional enrichment analysis

For graphically presenting gene ontology (GO) functions and Kyoto Encyclopedia of Genes and Genomes (KEGG) pathway enrichment analysis, specifically for genes with differential expression, the ggplot2 software package was utilized. The GO database offers insights into various gene-related aspects such as molecular functions, cellular components, and biological processes. Further, to delve into the possible functions of TIGD1, GO enrichment analysis was conducted using the ClusterProfiler software package.

### Immunoassay

In an effort to explore the potential immunoregulatory function of TIGD1 within tumor-infiltrating immune cells, patients were segregated into two groups based on their TIGD1 expression levels. These groups were classified as “high” and “low” TIGD1 expression, determined by the median value of TIGD1 expression. Utilizing the ESTIMATE algorithm, the study focused on analyzing the association between TIGD1 expression and the scores related to immune and stromal components. In assessing the extent of immune cell infiltration within lung adenocarcinoma (LUAD), the “Xcell” algorithm was utilized. This algorithm facilitated the determination of the presence of various immune cells, including CD8^+^ T cells, macrophages, and NKT cells. This research delved into significant genes associated with immune checkpoint blockade therapy, such as TNFRSF14, CD160, and TNFRSF45. It involved comparing the expression levels of 15 immune checkpoint blockade-related genes, like PDCD1, across patient groups categorized by low and high scores. Additionally, the study examined how TIGD1 expression levels, both high and low, correlate with the expression of key immune checkpoint blockade genes. The “Immuneeconv” R package was used to assess the expression of immune checkpoints and analyze their co-expression with TIGD1.

### Somatic mutation analysis

Our research focused on exploring somatic mutations across two risk groups, utilizing somatic alteration data obtained from the TCGA-LUAD cohort. This cohort forms part of the renowned TCGA dataset, which is extensively acknowledged in the field. A key parameter in our study is the Tumor Mutational Burden (TMB), which signifies the aggregate count of somatic, coding, nonsynonymous, and coding-translocation indel mutations for each megabase in the genome. TMB was computed with a threshold of 5% for detection. To precisely ascertain the quantity of somatic non-synonymous point mutations in each sample, the Maftools package in R was employed for summarizing the mutation data of key genes (12). This allowed us to analyze the tumor mutation load (TMB) in detail, providing crucial data for further tumor characterization studies.

### Drug sensitivity analysis

In our study, we analyzed drug sensitivity among high-risk patient groups using the half-maximal inhibitory concentration (IC50) data obtained from the Genomics of Drug Sensitivity in Cancer (GDSC) database, which is available at https://www.cancerrxgene.org.

### Cell culture

In this study, all cell lines were obtained from the Cell Bank of the Chinese Academy of Sciences (China). The non-small cell lung cancer (NSCLC) cell lines H1299, H292, and H1975 were maintained in RPMI-1640 medium (Gibco, United States), supplemented with 10% fetal bovine serum (FBS) (Excell, China). In contrast, the human lung normal epithelial cell line BEAS-2B and the NSCLC cell line PC-9 were grown in DMEM medium, also supplemented with 10% FBS (Excell, China). All cell lines were cultured at 37°C in a humidified atmosphere containing 5% CO_2_.

### Clinical tissue sample

Clinical tissue specimens were acquired from 10 NSCLC patients at The First Affiliated Hospital of Nanchang University. All related experiments received approval from the ethics committee of the hospital, and informed consent was obtained from all patients.

### RNA interference

For gene interference purposes, we obtained small interference RNA (siRNA) targeting the TIGD1 gene and control RNA from GenePharma Company (Shanghai, China). These siRNAs and control RNA were transfected into H1299 cells using the jetPRIMER^®^ transfection reagent from Polyplus Transfection, France, achieving optimal cell confluence of 30–50%. The specific sequences used to target the TIGD1 gene are provided in Table 1.

**Table 1:** TIGD1 gene siRNA sequence.

Gene name	Sequences (5′-3′)	Sequences (3′-5′)
TIGD1-804	GCGGCAAACAGUUAGCCAATT	UUGGCUAACUGUUUGCCGCTT
TIGD1-988	CCAGAACAAAGCCCUAACUTT	AGUUAGGGCUUUGUUCUGGTT
TIGD1-1170	CAGAAGCUUUAGCUAAGAUTT	AUCUUAGCUAAAGCUUCUGTT
TIGD1-1742	GCCAUGGAUAGUGAUGUCUTT	ACGUGACACGUUCGGAGAATT
Negative control	UUCUCCGAACGUGUCACGUTT	ACGUGACACGUUCGGAGAATT

### Quantitative real-time PCR

Total RNA was extracted from both cell and tissue samples utilizing Trizol reagent. Subsequently, this RNA was reverse transcribed into cDNA following the protocol of Prime Script RT Master Mix (Takara Biotechnology, Dalian, China). To measure gene expression levels, quantitative real-time PCR (qRT-PCR) was conducted with the Hieff UNICON^®^ Universal Blue qPCR SYBR Green Master Mix (YEASEN, Shanghai, China) and the ABI7500 System (Thermo Fisher, United States), following the manufacturers’ guidelines. The specific primer sequences used are listed in Table 2.

**Table 2:** Primers sequence.

Primer	Forward primer (5′-3′)	Reverse primer (3′-5′)
TIGD1	CAAATGGAGGGTTTGTGGCA	ACGAGTGAACCACAGAGACA

### Cell Counting Kit-8 assay

H1299 cells were plated at a concentration of 1,000 cells per well in 96-well culture plates. After allowing for overnight adhesion, these cells were subjected to treatment the next day. Following the manufacturer’s guidelines, cell viability was assessed using the Cell Counting Kit-8 (CCK-8) assay (Abmole, United States), at various time intervals (0 h, 24 h, 48 h, 72 h, 96 h). The viability was quantified by measuring the absorbance at 450 nm with a microplate reader (Thermo Fisher, United States).

### Plate clone formation assay

The cells were counted 24 h after treatment, and then added to each well of the 6-well plate. 4 mL medium of 10% FBS was inoculated with approximately 800 cells per well and immediately shaken. After approximately 10 days of incubation, the container was removed and cleaned three times with PBS. It was then fixed with ice Methanol for 10–20 min. After another round of cleaning, the cells were stained with crystal violet for 10–20 min and washed with distilled water. Finally, the biological imaging system was used to observe and count the cells.

### Wound-healing assay

The cell migration capability was assessed using the scratch wound-healing assay. Cells were allowed to grow until they reached 90% confluence in a 6-well plate. A wound was then created along the centerline of each well’s bottom using a 200-μL pipette tip. After thorough washing with phosphate-buffered saline, medium containing 1% FBS was added to each well.

### Western blotting

For protein samples, we used RIPA (Solaribio, China) to extract total protein from cells and tissue samples mentioned above on the ice. Then equal amount of proteins in each sample was separated by 10–12% SDS-PAGE (Servicebio, China) and transferred onto polyvinylidene fluoride membranes (Millipore, United States). The membrane was treated with a blocking buffer, which consisted of TBST mixed with 5% skim milk, and this process was conducted at room temperature for 1 h. We used TIGD1 (1:1000, 13833-1-AP, proteintech, China), AKT (1:1000, 10176-2-AP, proteintech, China), Phospho-AKT (1:1000, 28731-1-AP, proteintech, China), PI3K (1:1000, 20584-1-AP, proteintech, China), Phospho-PI3K (1:1000, 17366, Cell Signaling Technology, United States), GAPDH (1:100000, 60004-1-Ig, proteintech, China), Beta Actin (1:20000, 66009-1-Ig, proteintech, China) antibodies for incubation at 4°C overnight. Following three washes with TBST, the membrane was subjected to incubation with secondary antibodies. Subsequently, it was treated with enhanced chemiluminescence (ECL) (Easysee Western Blot Kit, China), and digital blot images were captured using an imaging system (Bio-Rad, United States).

## Results

### Expression of TIGD1 genes and functional enrichment analysis

**Figure 1:**
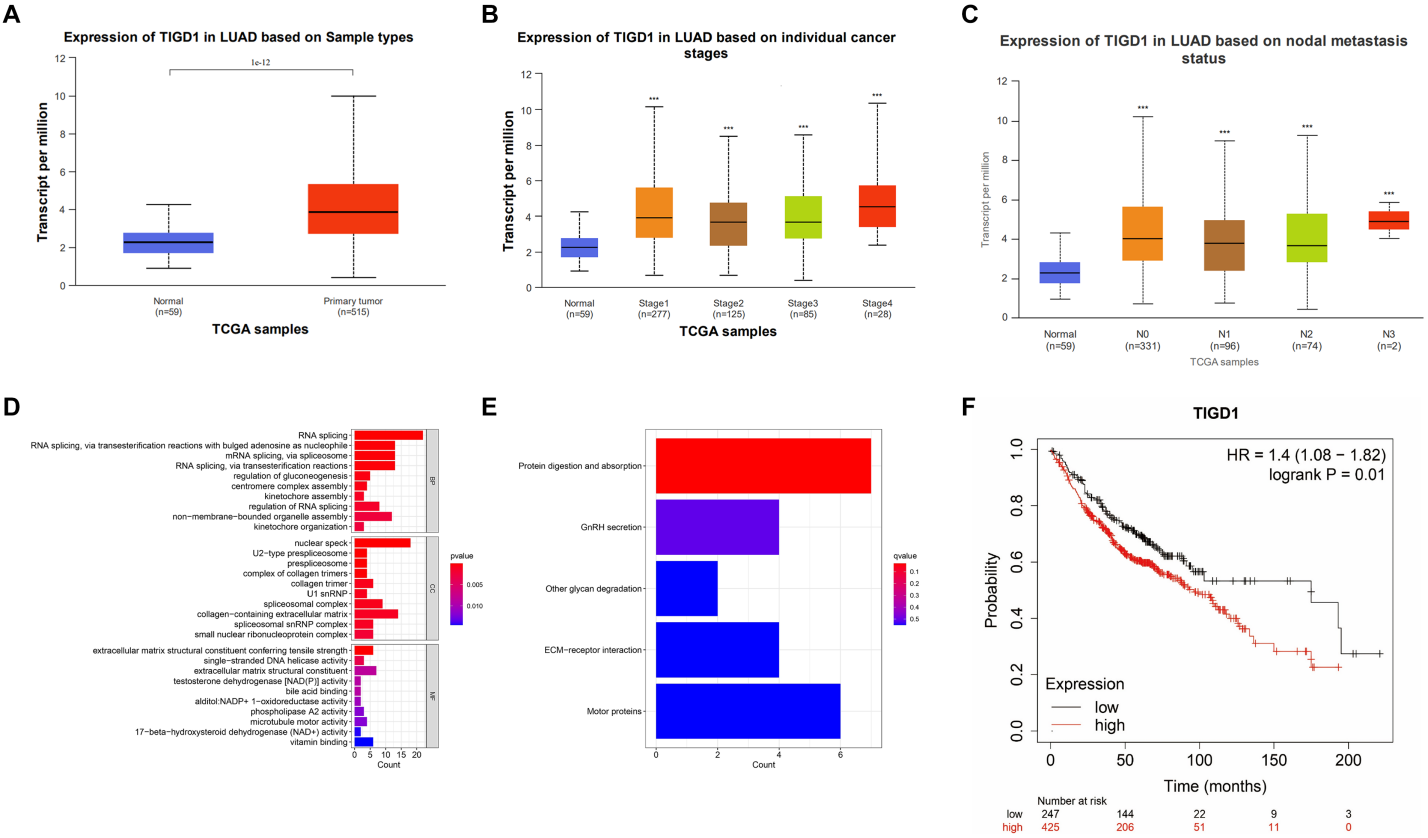
Integrated analysis of TIGD1 expression in lung cancer patients was conducted using the TCGA database. **(A)** The gene expression profiles and clinical data of 574 lung cancer patients were obtained through the TCGA database; **(B)** The relationship between TIGD1 expression and tumor grade in patients with lung cancer. The expression of TIGD1 is increased in patients with high-grade lung cancer (*p* < 0.001); **(C)** The relationship between TIGD1 expression and lymph node metastasis in patients with lung cancer. The expression of TIGD1 in N3 is higher than that in N2 and N1 stages (*p* < 0.001); **(D)** The GO enrichment analysis revealed that TIGD1 showed significant enrichment in RNA splicing, mRNA splicing, nuclear speckles, and extracellular matrix components; **(E)** KEGG enrichment analysis found that TIGD1 is mainly enriched in protein digestion and absorption, sex hormone secretion, and glycan degradation; **(F)** OS risk score between patients in the TIGD1 high expression group and low expression group. The results show that high TIGD1 expression is positively correlated with poor OS in lung cancer patients. ***p < 0.001.

From the TCGA database, we retrieved the gene expression profiles along with clinical data for 574 individuals diagnosed with lung cancer. Following the data integration, we examined the differential expression of TIGD1 between normal and tumor tissues. Compared with normal tissues, significantly higher expression of TIGD1 was found in human lung cancer tissues (*p* < 0.001) (Figure 1A). And it was found that the expression of TIGD1 is significantly increased in patients with high-grade lung cancer (*p* < 0.001) (Figure 1B). N3 expression is significantly higher than N1 and N2 expression in lymph node metastasis classification (*p* < 0.001) (Figure 1C). Enrichment analysis revealed that TIGD1 exhibited significant enrichment in RNA shearing, mRNA shearing, nuclear speckles, and extracellular matrix components. Additionally, it was also found to be enriched in processes such as protein digestion and absorption, sex hormone secretion, and glycan degradation (Figures 1D,E).

### TIGD1 high expression correlates with lower survival in NSCLC

Assessing OS risk scores showed a clear link between higher TIGD1 expression and reduced overall survival in lung cancer patients across different expression groups (*p* = 0.01) (Figure 1F).

### Analysis of immune microenvironment

**Figure 2:**
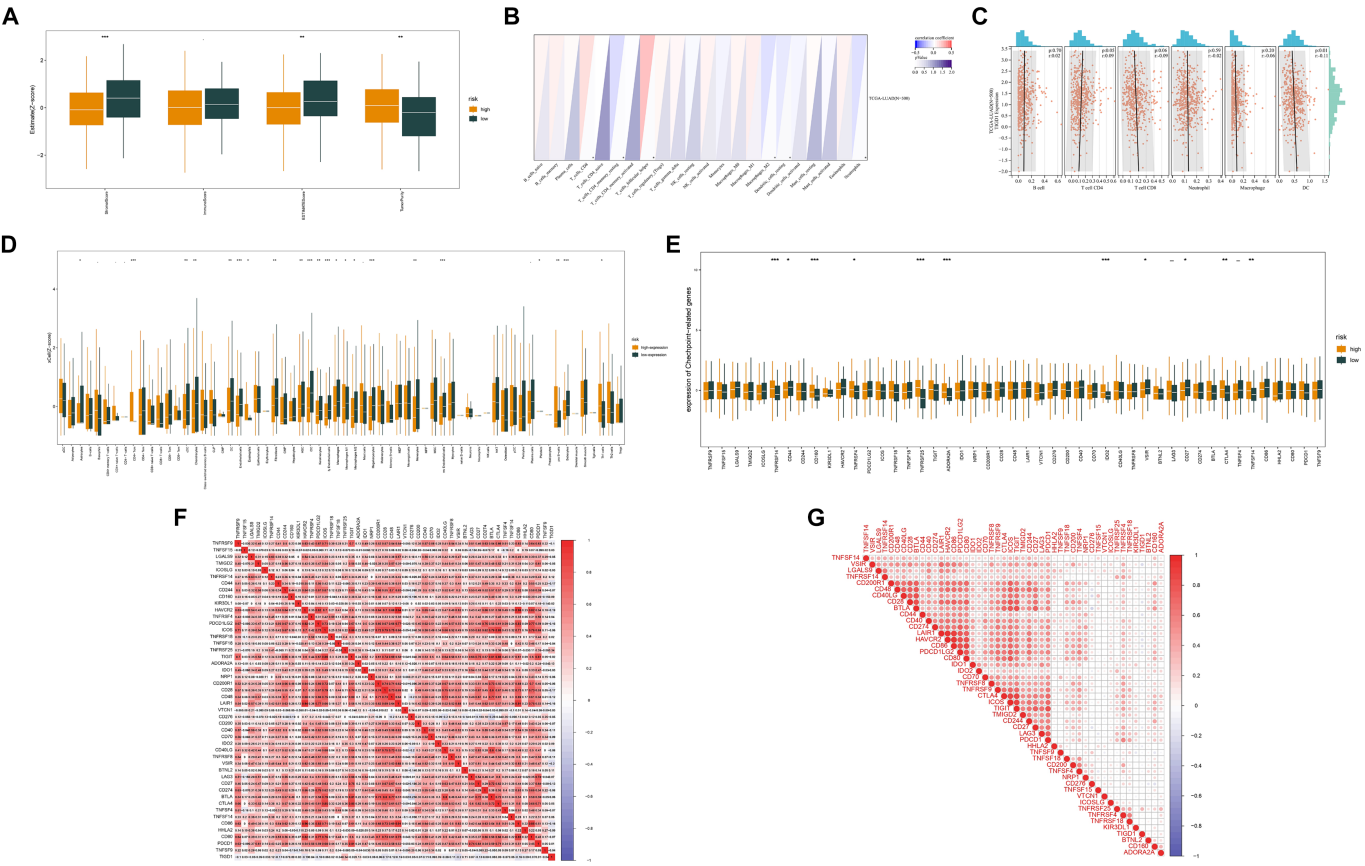
Analysis of the immune microenvironment. **(A)** Correlation analysis was conducted to examine the immune microenvironment of both the TIGD1 high-expression group and the low-expression group; **(B,C)** The relationship between TIGD1 expression and immune cells, TIGD1 expression is positively correlated with CD4 T cell infiltration (*r* = 0.09, *p* = 0.05), and negatively correlated with DC cell infiltration (*r* = −0.01, *p* = 0.01); **(D)** Analysis of immune function in TIGD1 high expression group and TIGD1 low expression group. The expression of CD4 cells is increased (*p* < 0.001), while endothelial cells, IDC cells, mv endothelial cells pro Reduced expression in B cells, Sebocytes cells (all *p* < 0.001); **(E)** Analysis of immune checkpoint proteins in the TIGD1 high expression group and low expression group revealed a significant increase in the expression of TNFRSF14, CD160, TNFRSF45, ADORA2A, and IDO2 (all *p* < 0.001), as well as CTLA4 and TNFSF14 (both *p* < 0.01); **(F,G)** Correlation analysis between TIGD1 and immunotherapy-related positive markers. *p < 0.05; **p < 0.01; ***p < 0.001.

Exploring the immune microenvironment uncovered notable variations in stromal cell expression, immune cell levels, and tumor purity between high and low TIGD1 expression groups (Figure 2A). TIGD1 expression shows a positive correlation with CD4 T cell infiltration (*p* < 0.05) and an inverse relationship with DC cell infiltration (*p* < 0.01) (Figures 2B,C). Analysis of immune function was conducted on two groups: the TIGD1 high expression group and the TIGD1 low expression group. The histogram (Figure 2D) reveals a significant correlation between TIGD1 expression level and immune cell expression. CD4 cell expression increased (*p* < 0.001), whereas the expression of endothelial cells, IDC cells, mv endothelial cells pro, B cells, and Sebocytes cells decreased (all *p* < 0.001). The analysis of immune checkpoints in the two groups revealed a significant increase in the expression of immune checkpoint proteins. These include TNFRSF14, CD160, TNFRSF45, ADORA2A, and IDO2 (all *p* < 0.001), as well as CTLA4 and TNFSF14 (all *p* < 0.01) (Figure 2E). And Correlation studies were performed to investigate the association between TIGD1 and markers positively related to immunotherapy. Findings indicated a positive correlation of TIGD1 with TNFRSF25, BTNL2, CD160, and ADORA2A, while an inverse relationship was noted with HAVCR2 (Figures 2F,G).

### Correlative analysis of tumor mutation burden

**Figure 3:**
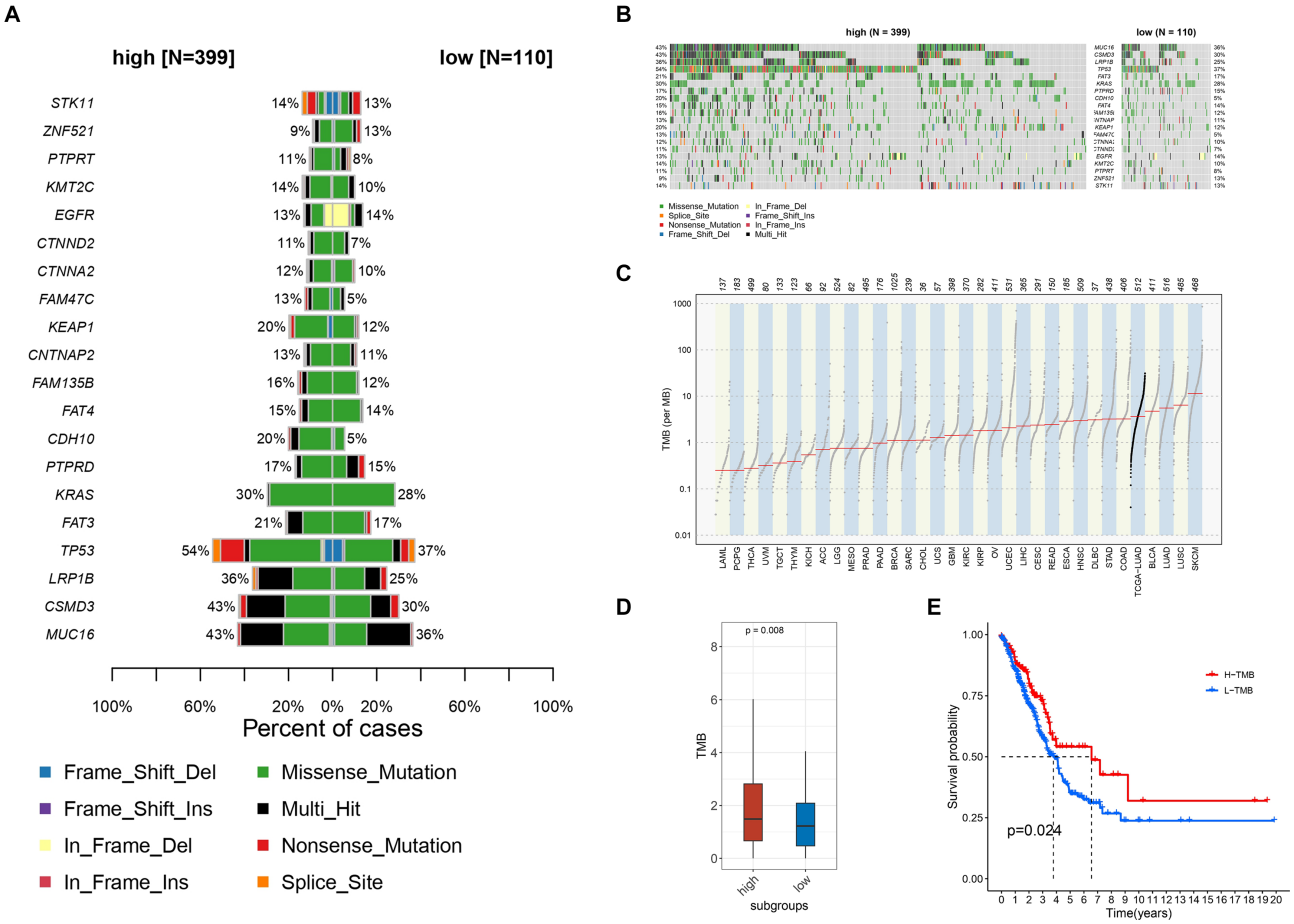
Relationship between different TIGD1 expression levels, tumor mutation burden, and patient survival outcomes. **(A,B)** Variation data of genes with high mutation frequency in TIGD1 high expression group and low expression group, there are significant differences in TMB; **(C,D)** There is a positive correlation between the expression level of TIGD1 and TMB (*p* = 0.008); **(E)** The Kaplan–Meier (KM) curve was utilized to conduct survival analysis on two groups: the high tumor mutational burden (TMB) group and the low TMB group. The survival outcomes of the low TMB group were found to be significantly poorer compared to those of the high TMB group (*p* = 0.024).

Data on gene mutations, particularly those occurring frequently, revealed significant differences between the groups with high and low expression levels of TIGD1 (Figures 3A,B). Our analysis revealed a significant positive correlation between the expression level of TIGD1 and TMB (*p* = 0.008) (Figures 3C,D). It was observed that the survival outcome for the group with low tumor mutation burden (TMB) was notably inferior in comparison to that of the group with high TMB (*p* = 0.024) (Figure 3E).

### Medication sensitivity analysis

**Figure 4:**
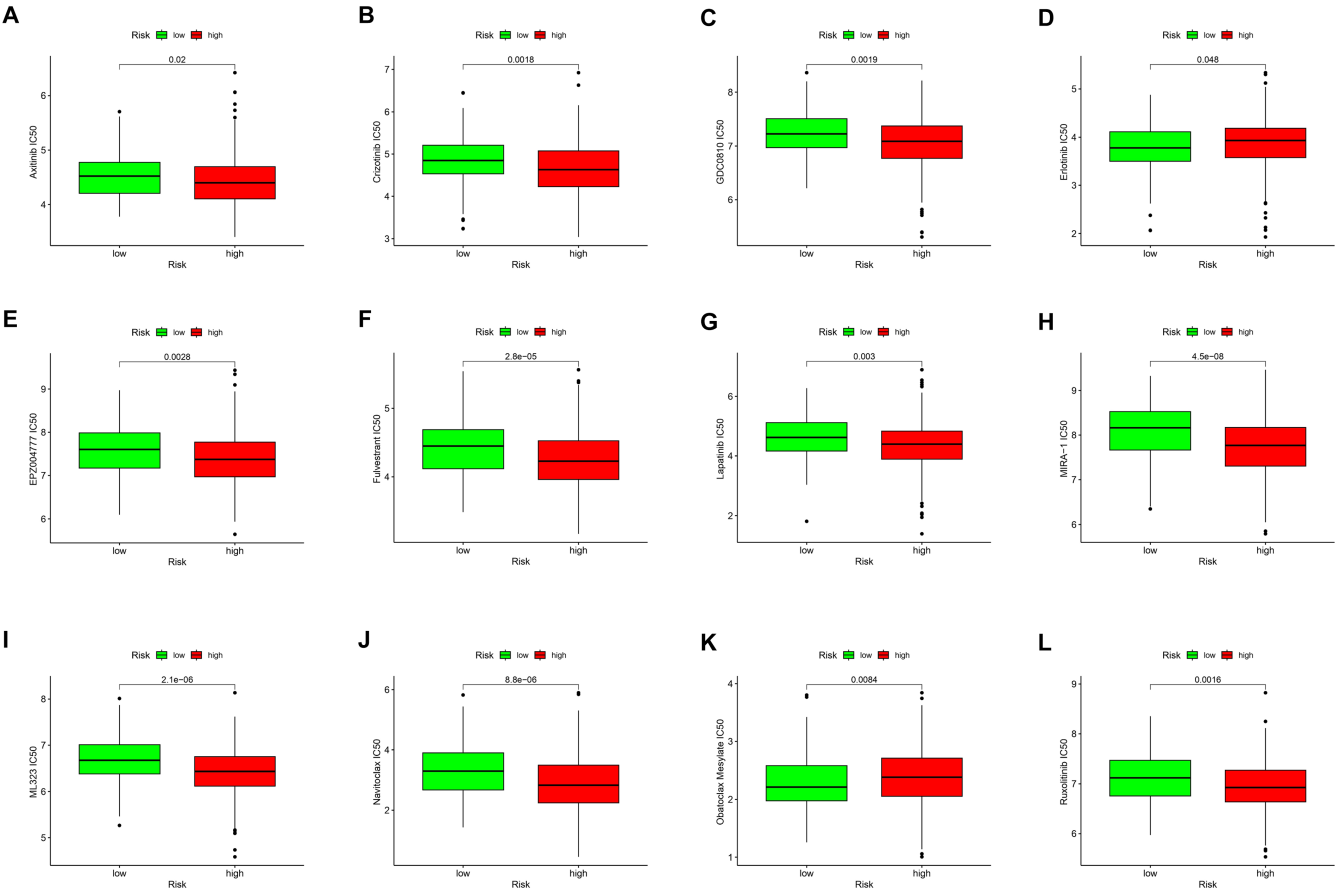
Drug sensitivity analysis. Drug sensitivity analysis of different drugs in TIGD1 high-risk group and low-risk group **(A–L)**, these drugs include: Axitinib IC50 (*p* = 0.02) **(A)**; Crizotinib IC50 (*p* = 0.0018) **(B)**; GDC0810 IC50 (*p* = 0.0019) **(C)**; Erlotinib IC50 (*p* = 0.048) **(D)**; EPZ004777 IC50 (*p* = 0.0028) **(E)**; Fulvestrant IC50 (*p* = 0.0028) **(F)**; Lapatinib IC50 (*p* = 0.003) **(G)**; MIRA-1 IC50 (*p* = 4.5e-0.08) **(H)**; ML323 IC50 (*p* = 2.1e-0.06) **(I)**; Navitoclax IC50 (*p* = 8.8e-0.06) **(J)**; Obatoclax Mesylate IC50 (*p* = 0.0084) **(K)**; Ruxolitinib IC50 (*p* = 0.0016) **(L)**.

As shown in Figures 4A–L, the estimated IC50s for 12 chemotherapy drugs (Axitinib, Crizotinib, GDC0810, Erlotinib, EPZ004777, Fulvestrant, Lapatinib, MIRA-1ML323, Navitoclax, Obatoclax, Mesylate, and Ruxolitinib) were higher in high-risk patients compared to those in the low-risk group. This suggests that low-risk patients may derive benefits from the use of these chemotherapy agents.

### TIGD1 gene is highly expressed in NSCLC tissues and cell lines

**Figure 5:**
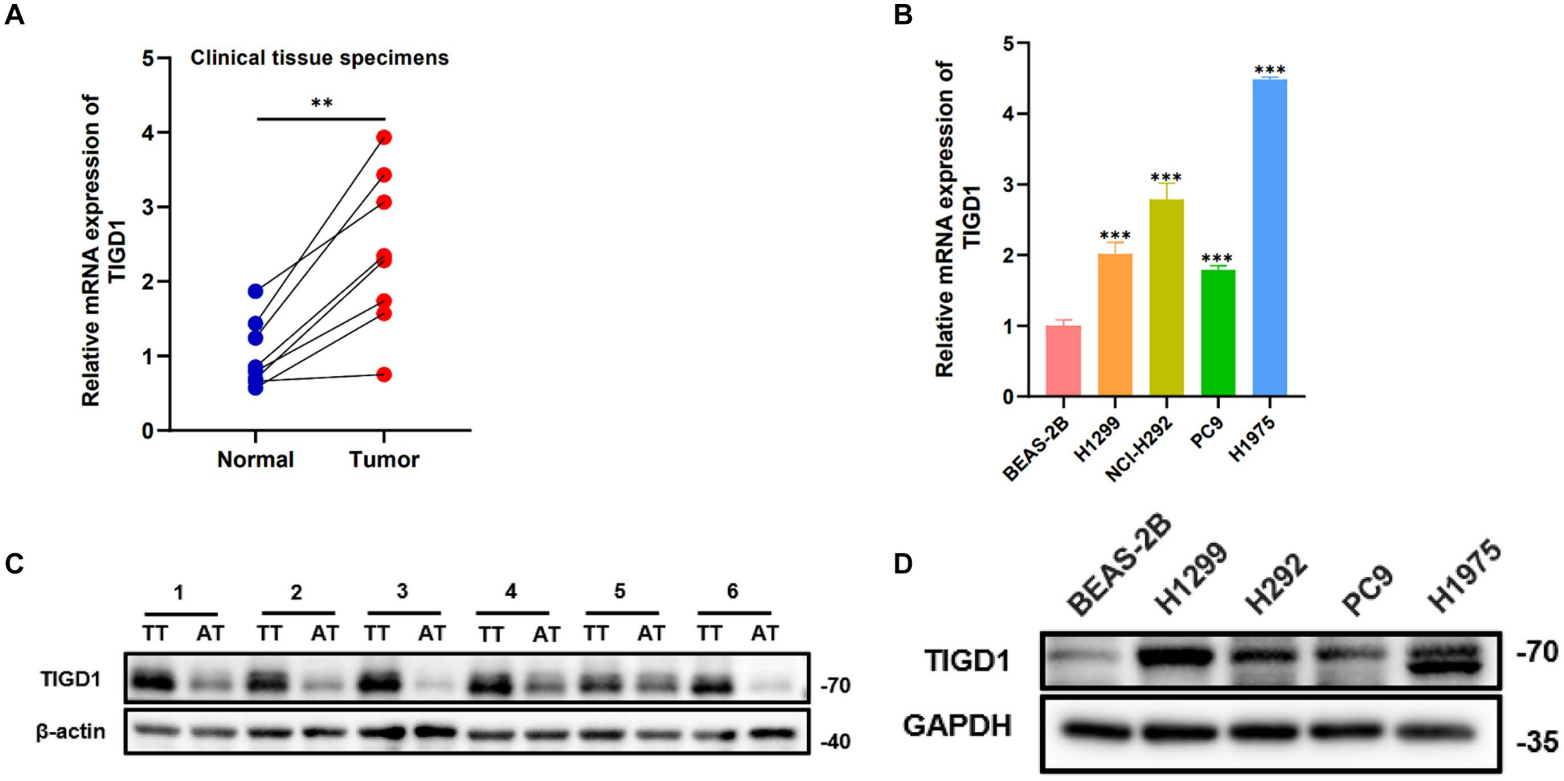
TIGD1 expression levels in lung cancer cell lines and tissues. **(A)** RT-qPCR detects the expression level of TIGD1 in lung cancer and paracancerous tissues; **(B)** The expression level of TIGD1 in BEAS-2B, H1299, H292, PC9, and H1975 cell lines was detected using RT-qPCR; **(C)** The protein expression level of TIGD1 in NSCLC and paracancerous tissues; **(D)** The protein expression level of TIGD1 in BEAS-2B, H1299, H292, PC9, and H1975 cell lines. **p < 0.01; ***p < 0.001.

The quantitative PCR (qPCR) results indicated a marked increase in TIGD1 expression in tumor tissues when contrasted with normal tissues (Figure 5A). Likewise, the level of TIGD1 expression in lung cancer cells was observed to be significantly elevated compared to that in normal cells (Figure 5B). Furthermore, Western blot analysis verified a significant increase in TIGD1 protein expression in both lung cancer cells and tumor tissues, in contrast to normal cells and neighboring non-tumorous tissues (Figures 5C,D).

### Knockdown of TIGD1 significantly inhibited the proliferation and migration of H1299 cells

**Figure 6:**
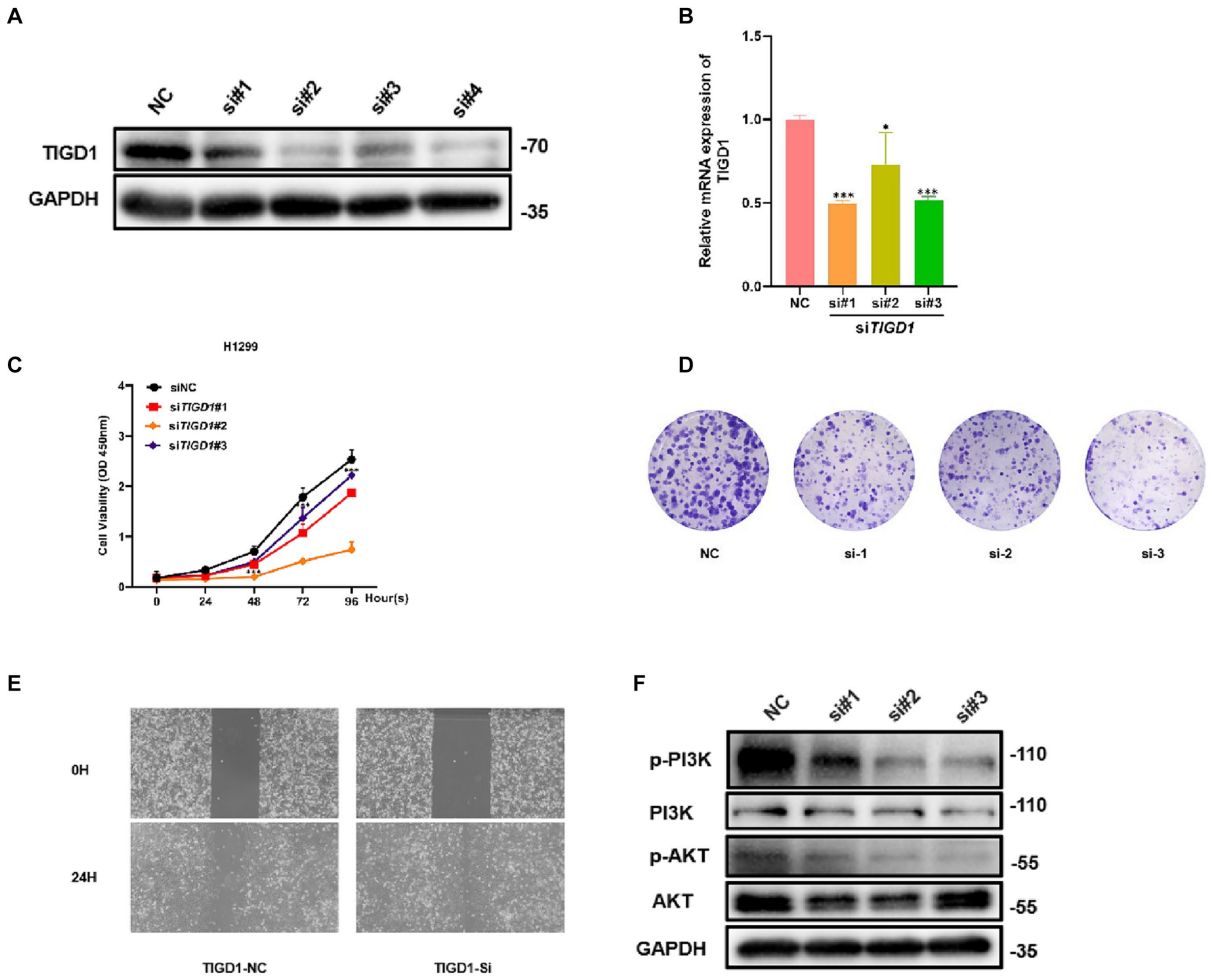
Functional experiments of TIGD1 in lung cancer cells. **(A)** Knockdown of TIGD1 protein expression levels was verified via Western Blot analysis; **(B)** Knockdown efficiency was verified using mRNA transcript levels quantification by qRT-PCR; **(C)** Cell Counting Kit-8 (CCK-8) assay showing cell viability; **(D)** colony-forming ability of lung cancer cell was determined using a plate colony-forming assay; **(E)** Wound-healing assay was used to test the effect of TIGD1 on lung cancer cell migration; **(F)** Western blotting detected p-PI3K, PI3K, p-AKT, and AKT protein expression levels. *p < 0.05; ***p < 0.001.

To study the function of TIGD1 in NSCLC, we knocked down TIGD1 by transfecting siRNA into H1299 cells. We measured knockdown levels using western blotting and qRT-PCR. When compared with the control group, the expression of TIGD1 in the inhibited group was significantly reduced (Figures 6A,B). Then, CCK-8 assay and plate clone-formation assay were used to assess cell proliferation ability. The results showed that downregulation of TIGD1 significantly reduced the viability of H1299 cells (Figures 6C,D). And we performed a wound-healing assay to evaluate the cell migration capacity. TIGD1 knockdown in H1299 cells resulted in a reduced motility (Figure 6E). The above results suggest that inhibiting TIGD1 gene expression can significantly inhibit the cell proliferation and migration *in vitro*.

### TIGD1 affects NSCLC proliferation by regulating PI3K/AKT pathway

To further explore the mechanism of TIGD1 promoting NSCLC proliferation, we examined the PI3K/AKT pathway after knockdown TIGD1 in H1299. The results from western blot suggest that after TIGD1 knockdown, the levels of p-Akt and p-PI3K were diminished, whereas the expression of AKT and PI3K remained largely unchanged when compared to the normal control group (Figure 6F).

## Discussion

Lung cancer remains one of the most prevalent types of cancer and is the foremost cause of cancer-related deaths worldwide (13). While there have been significant advancements in lung cancer therapy in recent years, it is important to address the persistent challenges of drug resistance, tumor recurrence, and metastasis (14). Yin et al. discovered that TIGD1 is linked to survival rates in cancer malignancies and plays a role in the regulation of cell-cycle progression in human cancers (7). Researchers have discovered that TIGD1 plays a role in the onset and progression of tumors like colorectal cancer and oral cancer (15, 16). However, the role of TIGD1 in lung cancer, especially in non-small cell lung cancer, has not been extensively explored in prior research.

In this study, we sourced clinical data and gene expression profiles of lung adenocarcinoma from a variety of databases, including The Cancer Genome Atlas (TCGA), UCSC XENA, and the Gene Expression Omnibus (GEO). Through advanced data analysis techniques, we uncovered a significant correlation between TIGD1 expression and key factors such as clinical characteristics, the immune microenvironment, tumor mutational burden (TMB), and drug sensitivity. Notably, TIGD1 expression was found to be elevated in lung cancer tissues compared to normal counterparts, particularly in patients with advanced-stage lung cancer and lymph node metastasis. Furthermore, patients with higher TIGD1 expression exhibited increased overall survival (OS) risk scores compared to those with lower expression levels, suggesting that TIGD1 may act as an oncogene in cancer progression.

It was also observed that TIGD1 expression positively correlates with CD4^+^ T cell infiltration and negatively with DC cell infiltration. Furthermore, the expression level of TIGD1 was found to be associated with the sensitivity of different drugs. These findings suggest that targeted immunotherapy could be achieved by modulating TIGD1. Consequently, the expression level of TIGD1 could serve as a valuable indicator for guiding clinical medication. Within the cytoplasm, the PI3-K complex is composed of a regulatory component weighing 85 kDa and a catalytic subunit of 110 kDa (17). Akt, also referred to as protein kinase B (PKB), is a serine/threonine kinase with a molecular weight of 57 kDa. It plays a crucial role in promoting cell survival in response to growth factor stimulation (18). The PI3-K/AKT signaling pathway is well-known and extensively acknowledged within the realm of cellular biology (19). Existing studies indicate the pivotal role of the PI3K/Akt pathway in facilitating the progression and proliferation of lung cancer (20, 21). Additionally, it has been found that suppression of this pathway significantly reduces the migration and invasion of non-small cell lung cancer (NSCLC) cells (22). In our research, we explored the relationship between TIGD1 and the PI3K/AKT signaling pathway. Western blot analysis revealed a decrease in p-Akt and p-PI3K levels following TIGD1 knockdown. This suggests that TIGD1 may influence NSCLC proliferation through modulation of the PI3K/AKT pathway.

In conclusion, our findings suggest that TIGD1 holds significant potential as a diagnostic and prognostic marker in lung cancer, offering a promising target for the development of targeted therapies, including those based on immunotherapy and specific drugs. Additionally, our results hint at TIGD1’s mechanism of action being linked to the regulation of the PI3K/AKT signaling pathway. Further research is necessary to uncover additional mechanisms and validate TIGD1’s role in cancer progression and treatment.

## Data availability statement

The datasets presented in this study can be found in online repositories. The names of the repository/repositories and accession number(s) can be found in the article/Supplementary material.

## Ethics statement

The studies involving humans were approved by the Ethics Committee of the First Affiliated Hospital of Nanchang University. The studies were conducted in accordance with the local legislation and institutional requirements. The participants provided their written informed consent to participate in this study.

## Author contributions

LX: Data curation, Formal analysis, Investigation, Methodology, Software, Supervision, Writing – original draft. ZY: Data curation, Investigation, Methodology, Software, Writing – original draft. MX: Data curation, Formal analysis, Software, Writing – original draft. GW: Formal analysis, Investigation, Methodology, Software, Writing – original draft. YM: Project administration, Software, Validation, Writing – original draft. YY: Project administration, Visualization, Writing – review & editing. JT: Funding acquisition, Resources, Supervision, Visualization, Writing – review & editing.

## Footnotes

^1^ http://ualcan.path.uab.edu

^2^ http://kmplot.com/analysis/
