# Supplementary material for: Bioinformatics analysis and experimental verification of TIGD1 in non-small cell lung cancer
Source: Front Med (Lausanne). 2024 Apr 8;11:1374260. doi: 10.3389/fmed.2024.1374260 (PMC11034383; doi:10.3389/fmed.2024.1374260)
Supplement: Supplementary file 1 [file Data_Sheet_1.PDF]

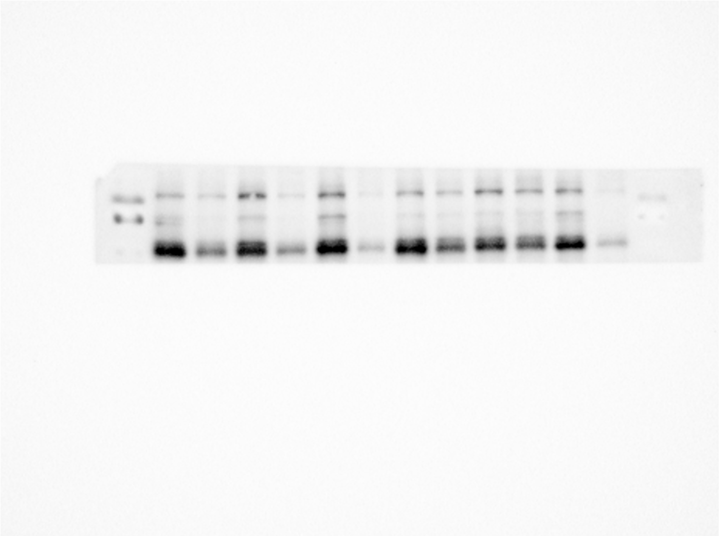

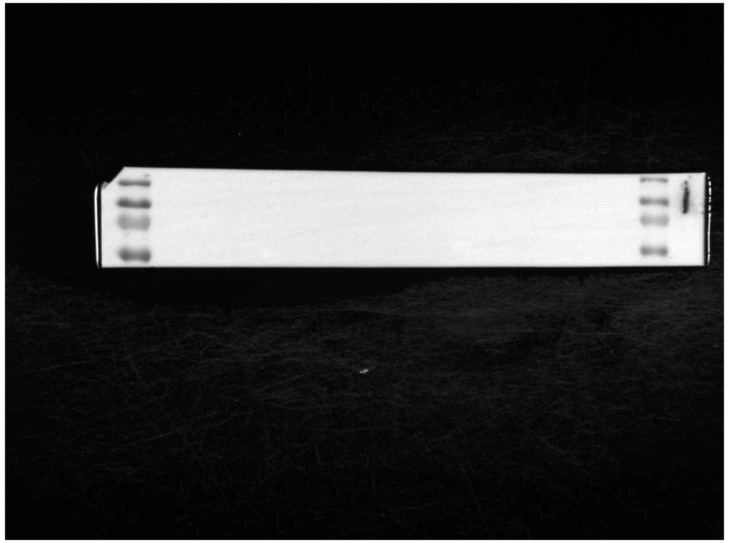

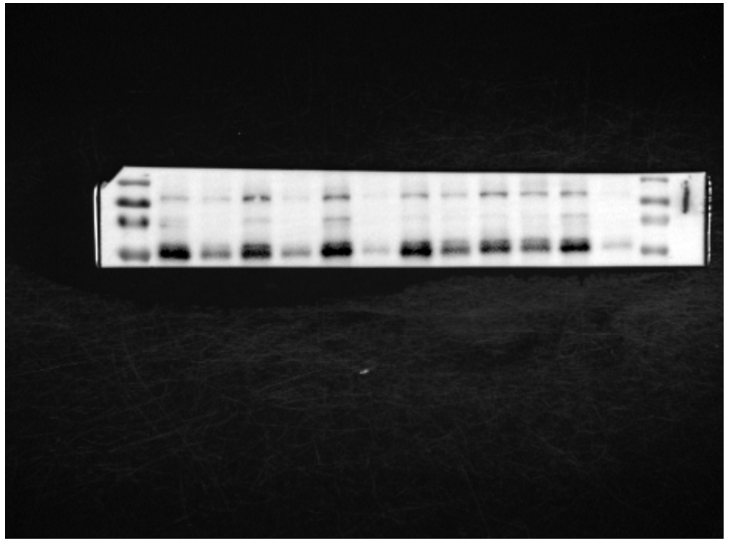

Figure 5C TIGD1
The protein expression level of TIGD1 in NSCLC and paracancerous tissues;
From left to right,
every two lanes from left to right are the same NSCLC patient’s cancer and paracancerous tissue

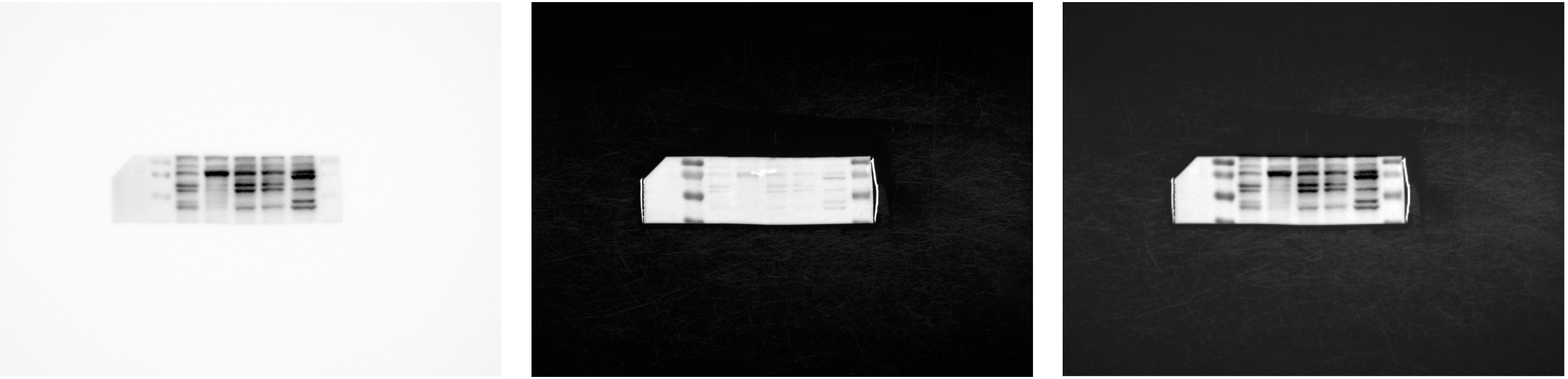

Figure 5D TIGD1

The protein expression level of TIGD1 in BEAS-2B, H1299, H292, PC9, and H1975 cell lines;

From left to right,

Lane 1= The protein expression level of TIGD1 in BEAS-2B;

Lane 2= The protein expression level of TIGD1 in H1299;

Lane 3= The protein expression level of TIGD1 in H292;

Lane 4= The protein expression level of TIGD1 in PC9;

Lane 5= The protein expression level of TIGD1 in H1975.

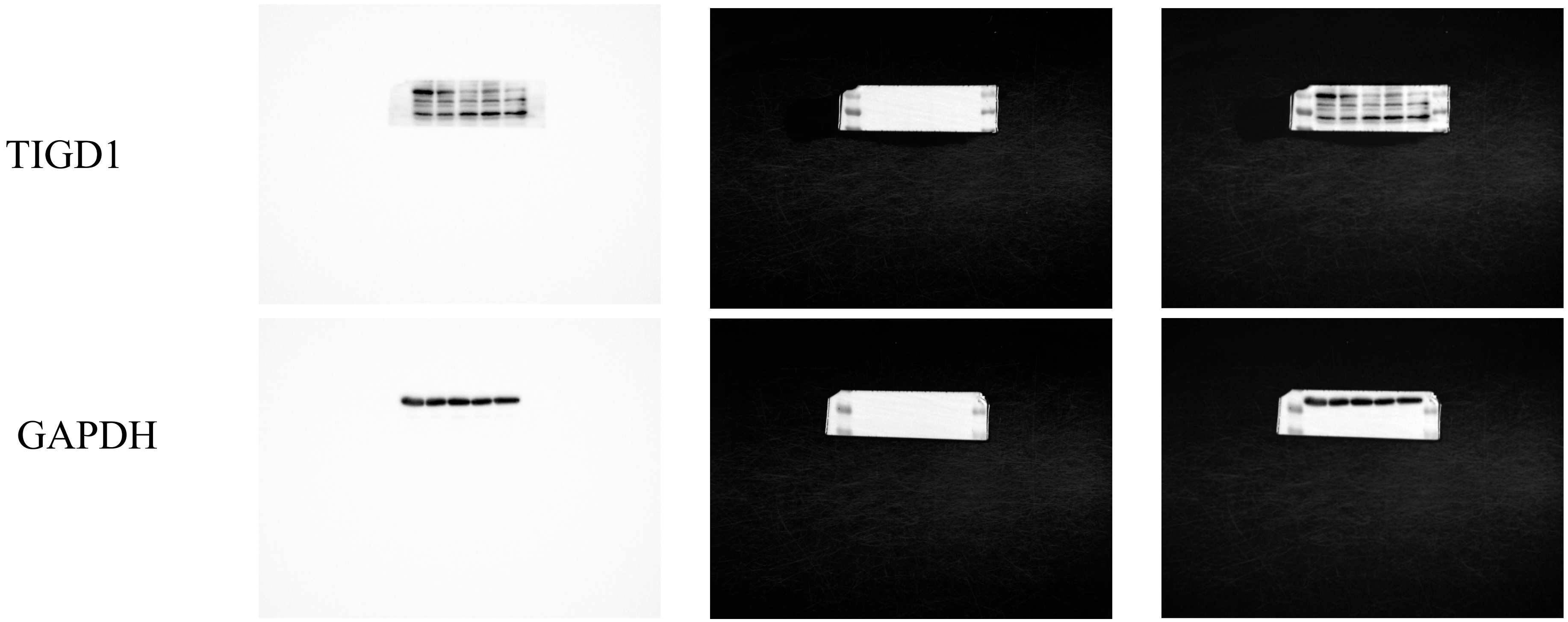

Figure 6A TIGD1,GAPDH
The protein expression level of TIGD1 after knockdown TIGD1
From left to right,
Lane 1= si-TIGD1-NC ;
Lane 2= si-TIGD1-1;
Lane 3= si-TIGD1-2;
Lane 4= si-TIGD1-3;
Lane 5= si-TIGD1-4;

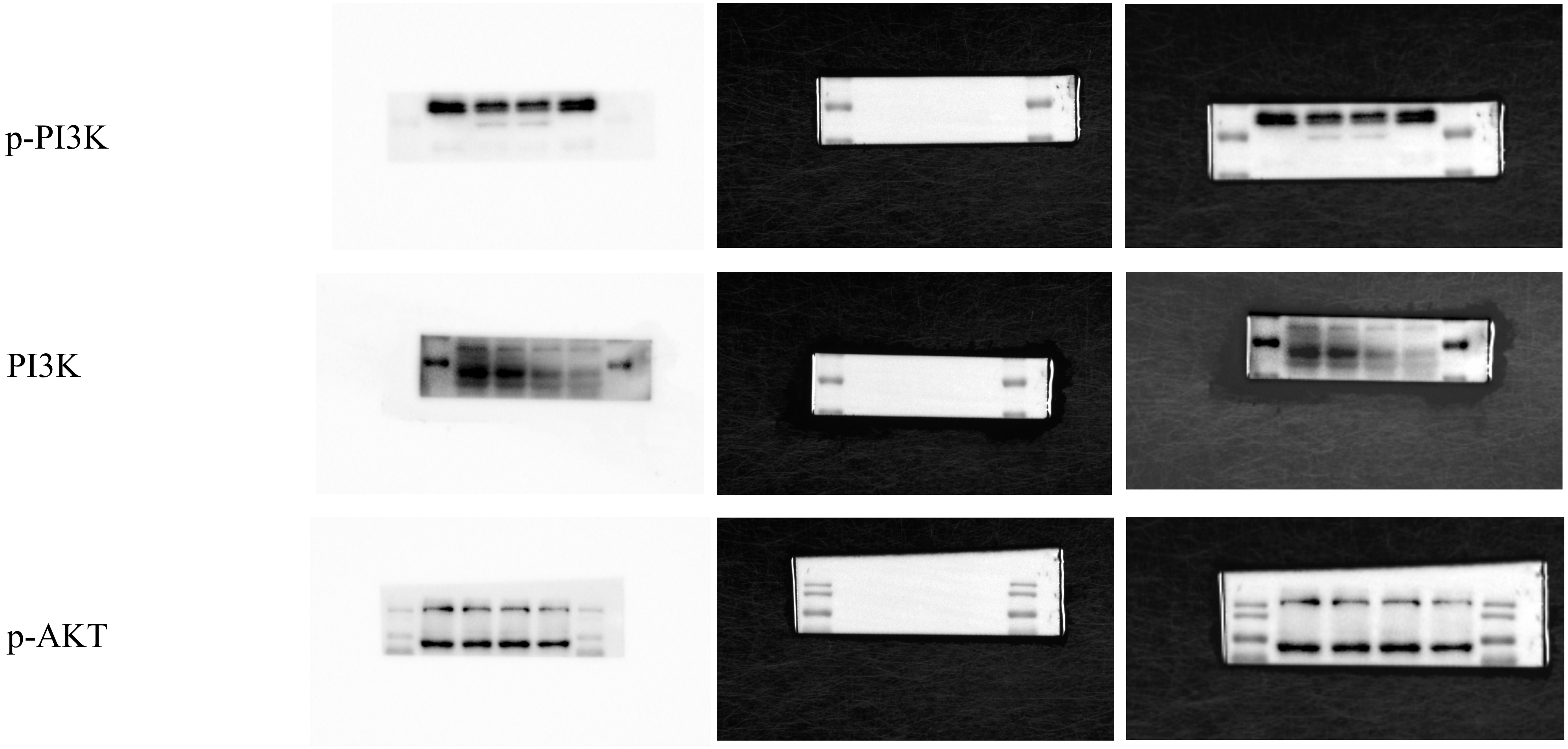

Figure 6F p-PI3K,PI3K,p-AKT,AKT,GAPDH
From left to right,
Lane 1= si-TIGD1-NC ;
Lane 2= si-TIGD1-1;
Lane 3= si-TIGD1-2;
Lane 4= si-TIGD1-3

AKT

GAPDH

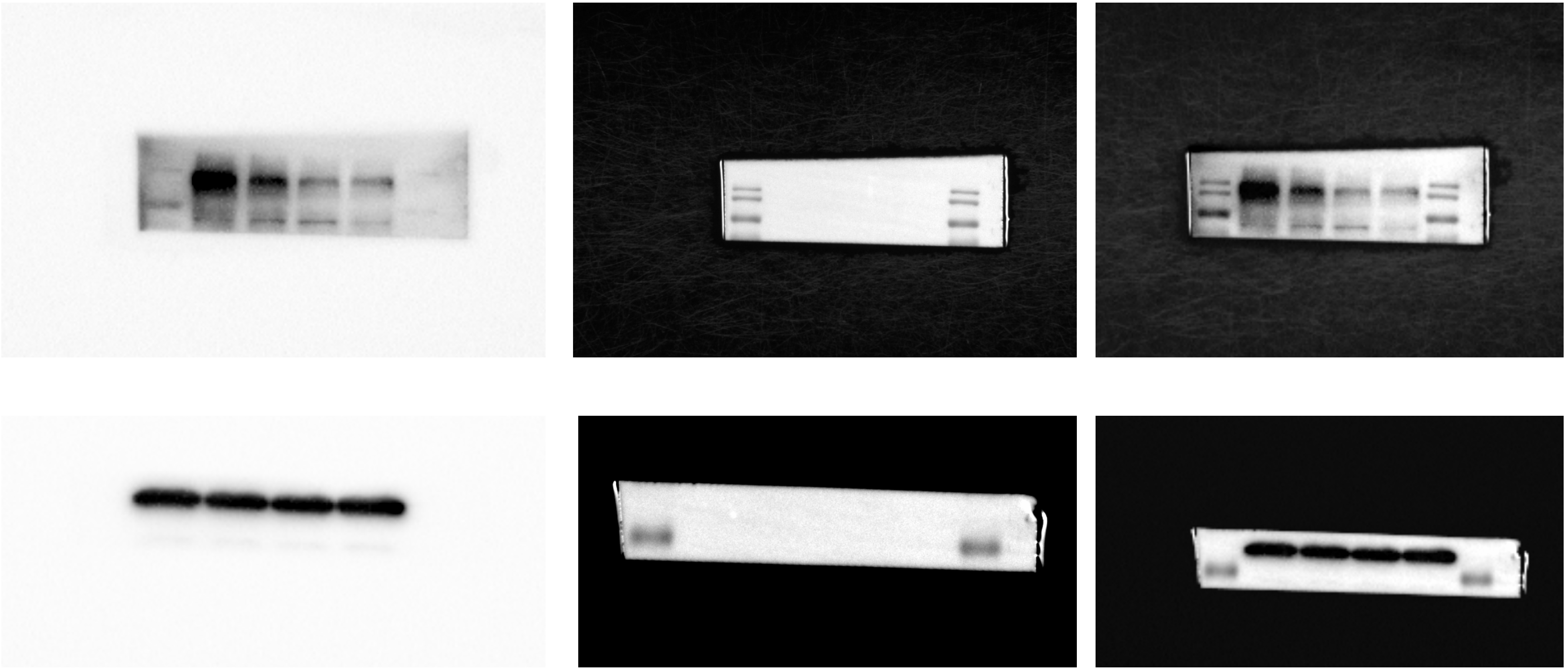

Figure 6F p-PI3K,PI3K,p-AKT,AKT,GAPDH
From left to right,
Lane 1= si-TIGD1-NC ;
Lane 2= si-TIGD1-1;
Lane 3= si-TIGD1-2;
Lane 4= si-TIGD1-3
